# Infrared Thermographic Evaluation of Exothermic Temperature Changes During the Setting Reaction of Six Dental Impression Gypsums: An In Vitro Linear Regression Model

**DOI:** 10.3390/dj14090582

**Published:** 2026-09-10

**Authors:** Franco Mauricio, Cesar Mauricio-Vilchez, Luzmila Vilchez, Mesias Rojas, Diego Galarza-Valencia, Fran Espinoza-Carhuancho, Frank Mayta-Tovalino

**Affiliations:** 1 Faculty of Dentistry, Research, Innovation and Entrepreneurship Unit, Universidad Nacional Federico Villarreal, Lima 15088, Peru; 2 Research Group on Neuroscience, Metabolism, Clinical and Health Efficacy (NEMECS), Universidad Científica del Sur, Lima 15081, Peru; 3 Vicerrectorado de Investigación, Universidad San Ignacio de Loyola, Lima 15024, Peru

**Keywords:** dental gypsum, infrared thermography, in vitro, thermal behavior

## Abstract

**Objective:** To evaluate surface temperature changes during the setting reaction of six dental gypsums using infrared thermography. **Methods:** This experimental in vitro study evaluated six dental gypsum materials randomly allocated into six groups: three Type IV materials (Elite Rock, Dürum, and Velmix) and three Type III materials (Elite Model, Pentadur, and Duromix). Specimens were manually mixed for a few minutes using a gypsum spatula and a rubber mixing bowl and subsequently formed in triangular stainless-steel molds with a 2 cm base and 2 cm equal sides. Thermographic evaluation was performed using a Fluke TiS55+ infrared camera. Surface temperature measurements were recorded for each specimen at baseline and at 10 and 25 min. **Results:** Distinct thermal profiles were observed among the dental gypsum materials. Compared with the baseline, Type IV dental gypsum showed an increase in temperature of 1.05 °C at 10 min (95% confidence interval [CI]: 0.23–1.87; *p* = 0.012) and 2.46 °C at 25 min (95% CI: 1.64–3.28; *p* < 0.001). Compared with Elite Rock, the reference material, Dürum and Velmix showed significantly lower temperatures, with differences between −2.94 °C and −2.47 °C, respectively (both *p* < 0.001). Type III dental gypsum showed an increase in temperature of 6.16 °C at 10 min (95% CI: 5.50–6.82; *p* < 0.001), followed by an increase of 1.76 °C at 25 min (95% CI: 1.10–2.42; *p* < 0.001). **Conclusions:** Type III dental gypsums exhibited marked early increases in temperature, whereas Type IV dental gypsums showed more gradual and material-specific thermal patterns. These findings demonstrate differences in the thermal behavior of the evaluated dental gypsum materials during the setting reaction and support further investigation into the relationship between thermal behavior and other physicochemical properties, including dimensional stability and material performance.

## 1. Introduction

Dental gypsum materials have long been used in dentistry for the fabrication of casts that reproduce the anatomical structures of the oral cavity. These casts remain essential for various diagnostic and restorative procedures, including the fabrication of dental prostheses and restorations [1,2,3,4]. The accuracy of the resulting restoration depends, among other factors, on the appropriate selection of the impression material and dental gypsum used for cast fabrication [5,6]. Although digital technologies have increasingly transformed dental workflows, conventional gypsum-based casts continue to represent a reliable method for obtaining physical models in several clinical and laboratory applications [7,8].

Calcium sulfate hemihydrate constitutes the principal component of dental gypsum materials. When mixed with water, calcium sulfate hemihydrate undergoes a hydration reaction that leads to the formation of calcium sulfate dihydrate, a process that underlies the setting behavior and physical properties of the material [9,10]. Dental gypsum products are classified into Types I–V according to established standards, including those of the American Dental Association and the International Organization for Standardization (ISO 6873) [11,12].

The selection of appropriate dental gypsum material depends on the intended clinical or laboratory application. Type III dental gypsum is commonly used for fabricating diagnostic models [13]. In contrast, Type IV and Type V dental gypsum materials are routinely used for the fabrication of definitive casts, particularly for procedures involving indirect restorations such as inlays, bridges, and crowns [14].

During the hydration process of dental gypsum, the formation and continuous growth of calcium sulfate dihydrate crystals may result in volumetric expansion of the material [15,16,17]. Previous studies have indicated that dental gypsum materials with a lower expansion coefficient may provide greater dimensional accuracy and stability, thereby contributing to the accurate reproduction of surface details [14,18].

The properties and structural integrity of dental gypsum casts may be influenced by factors related to material handling [19] and the drying process. Consequently, different temperature-based drying methods have been investigated to assess their effects on the properties of the resulting casts [20,21,22,23,24]. Characterizing the thermal profile during the setting reaction may therefore contribute to a better understanding of the hydration process.

Since the setting and drying processes of dental gypsum materials are associated with temperature variations, noninvasive thermal imaging techniques, such as infrared thermography, may be useful for characterizing surface temperature changes during the setting reaction. Infrared thermography enables noncontact assessment of the surface thermal profile over time. However, the available scientific literature on the thermal behavior of dental gypsum materials during setting remains limited. Therefore, this study aimed to evaluate surface temperature changes during the setting reaction of different dental gypsum materials using infrared thermography and linear regression analysis.

## 2. Materials and Methods

### 2.1. Ethics

This study was conducted in full compliance with the ethical principles of the Federico Villarreal National University and was approved by its Ethics Committee under Resolution No. 001-2026.

### 2.2. Study Design

This was an in vitro experimental study designed to evaluate temperature variation in six dental impression gypsum types (Type III and Type IV) using infrared thermography. This in vitro study was conducted in strict adherence to the CRIS Guidelines (Checklist for Reporting In vitro Studies) [25].

### 2.3. Sample Size

The sample size included in this study was based off pilot data collected at the beginning of this study, where we found the average value(s) and standard deviation(s) from a sample’s temperature deviation to determine the variability of that sample. Using Stata (version 17.0) as the statistical analysis software to calculate a mean comparison based upon a statistical power of 80% and α = 0.05, the total sample size for this research project is 60 specimens to be split evenly into six experimental groups (3 Type III and 3 Type IV Dental gypsum), meaning that there will be 10 specimens in each group.

### 2.4. Allocation

The specimens were distributed into six groups (3 for Type IV dental gypsum Elite Rock, Dürum, and Velmix and Type III dental gypsum Elite Model, Pentadur, and Duromix) using random allocation to maximize methodological integrity and reduce potential bias in all aspects of this experiment. For each group, there were 10 specimens, equating to 60 overall samples. At three-time intervals, baseline, 10 min, and 25 min after initial placement, all specimens were assessed on a systematic basis. This method of allocation allowed for an equitable comparison of the two different types and brands of gypsum with respect to each other, providing consistent comparison and repeatability of testing among the thermographic assessments.

In this in vitro experimental study, no random allocation procedure was used due to group pre-definition based on the type of dental gypsum under investigation (six groups). Rather, each specimen assigned to the appropriate group. Also, all specimens were measured for temperature at baseline, at 10 min, and after 25 min following an established schedule that included specific measurement conditions that were identical for all experimental groups.

### 2.5. Selection Criteria

The study only included Dental Impression Gypsum that was commercially manufactured and classified as Type III and IV. Groups for Type IV were Elite Rock, Duerum, and Velmix. For Type III, the groups were Elite Model, Pentadur, and Duromix. Testing specimens were prepared by the same manufacturer and under standard laboratory conditions. All required steps were taken to create comparable results. Any dental impression gypsum that was past its expiration date, improperly stored, or had serious surface defects were not considered for this research because these conditions would affect the reliability of the thermographic assessments.

### 2.6. Sample Preparation

Before preparing specimens, the operator underwent a process to standardize their manual mixing technique so that all specimens were prepared consistently. This involved reviewing the guidelines provided by manufacturers, and practicing using standardized ratios of powder-to-water, amounts of time to mix and the technique used for mixing (spatulation). The same individual prepared all specimens with identical tools and for the same duration of 1 min using a consistent rhythm and spatulation technique for all groups. Consequently, the mixing times for each of the materials were varied and were not set experimentally for a standardized fixed amount of time; rather, each of the materials was mixed manually in accordance with the manufacturer’s recommended mixing instructions (as described in Table 1). Baseline, 10 and 25 min evaluation points were not indicative of the time of mixing but rather represented the elapsed setting times after the preparation of the specimen. The 10 and 25 min periods were chosen to provide a means of tracking surface phenomenon occurring at the midpoint and end of the setting reaction using infrared thermography. To this end the thermal analysis was conducted at three pre-set points of the complete setting process, while the mixing and preparation were performed in accordance with the specific instructions provided by each specific dental gypsum manufacturer (Table 1).

The Type III dental gypsum samples included Elite Model, Pentadur, and Duromix; Type IV dental gypsum included Elite Rock, Dürum, and Velmix. The samples were prepared using a dental gypsum spatula and a rubber mixing bowl under the careful supervision of an operator skilled in dental materials to limit the potential variability between samples. The mixing guidelines for dental gypsum were strictly adhered to, including mixing for 1 min in an uninterrupted, rapid, and uniform manner by hand (to the consistency of creamy and blended). The samples were mixed in stainless steel triangular-shaped molds with bases of 2 cm and equal lengths to ensure uniform shape of all samples. All samples were prepared in the Laboratory of Operative Dentistry and Biomaterials at the Universidad Nacional Federico Villarreal (UNFV) under environmental conditions maintained at 23 ± 1 °C.

Evaluation times were chosen to represent all the important stages of the Gypsum Setting Reaction: Baseline evaluations indicated where gypsum was at thermal equilibrium before the beginning of the exothermic process of hydration, 10 min was chosen as the period in which most of the heat generated (exothermically) will occur, 25 min was selected because this allows for the majority of the hydration reaction to occur and the material to be thermally stabilized and consequently enables the comparison of the remaining temperature produced by each of the gypsum brands.

The preparation of all dental gypsum specimens was performed using stainless steel molds with equivalent dimensions of approximately 2 cm per side, resulting in uniform equilateral triangular shapes being prepared for each specimen. Each mold has an interior depth of approximately 1.5 cm and a metallic wall thickness of approximately 1.5 mm. The freshly mixed dental gypsum was poured into each of the molds to fill up their standardized volume, thus providing uniform specimens within each test group. All thermographic measurements were made while the dental gypsum was still contained within the stainless-steel molds during the setting process. This standardized configuration was established in order to reduce variabilities resulting from differences in the specimen’s shape and handling procedures; and to provide consistent, controlled conditions for heat release during thermographic imaging.

### 2.7. Thermographic Evaluation

Variations in temperatures were determined using a Fluke TiS55+ infrared thermal imaging camera (Fluke, Everett, WA, USA). A specific distance of 20 cm (20 cm) was maintained while acquiring images from each of the test samples; hence, the image acquisition techniques were the same. The following specifications were used on the Fluke TiS55+ camera: infrared resolution of 256 × 192 pixels. The camera’s spatial resolution (IFOV) was 1.91 Mrad. Images were acquired at a fixed distance of 20 cm perpendicular to the specimen. The Fluke TiS55+ infrared thermal imaging camera (Fluke, Everett, WA, USA) was calibrated before testing by a professional engineer to assure precise and repeatable results. Thermography was performed once at baseline and again after 10 (10) and 25 (25) minutes in the controlled laboratory environment (ambient temperature of 23 °C +/) at one degree Celsius (±1 °C), and all tests were performed under standardized illumination to allow consistent exposure to the test subjects and minimize any possible interference from external ambient conditions. The thermographic images were evaluated and processed using the SmartView Classic 4.4 Software Package, which generated and allowed the precise extraction of temperature data and a graphical representation of the thermal gradient. Thermal images were obtained using a thermal sensitivity of <0.05 °C, spectral range 7.5–14 μm, accuracy ±2 °C, emissivity set at 0.95, positioned 20 cm perpendicular to the specimen. Emissivity was fixed at 0.95 according to previous studies on gypsum materials. The temperature value used for analysis corresponded to the central point of each specimen, which was defined as the region of interest (ROI) to standardize measurements across all samples and ensure reproducibility. For each thermographic image, the temperature measurement was obtained from a single standardized point located at the geometric center of the specimen.

### 2.8. Statistical Analysis

Stata 17.0 (StataCorp, College Station, TX, USA) was used to perform the statistical analysis. Data were assessed using a Shapiro–Wilk Test for Normality. Most datasets failed to satisfy the assumption of normality (*p* < 0.05) so nonparametric statistical tests were used to compare temperature values among dental gypsum materials at each evaluation time point (10 and 25 min). Intergroup comparisons between temperature values among different dental gypsum types at each evaluation time point were assessed using the Kruskal–Wallis test for non-parametric testing, followed by appropriate post hoc pairwise comparisons for groups that had statistically significant differences.

To assess surface temperature over periods of time and the differences between dental gypsum types, separate linear regression analyses for dental gypsum types III and IV were conducted. Temperature (°C) was the dependent variable, and evaluation period (baseline vs. evaluation points) was the categorical independent variable to assess change in surface temperature compared to baseline values between dental gypsum types over time. Material type was a second categorical independent variable, where Elite Rock served as the base material for the Type IV model and Elite Model served as the base material for the Type III model. Provided in the linear regression analysis were regression coefficients (β), intercepts, 95% confidence intervals (CIs), and corresponding *p*-values. All statistical tests were two-tailed, and statistical significance was established at *p* < 0.05.

## 3. Results

**Figure 1:**
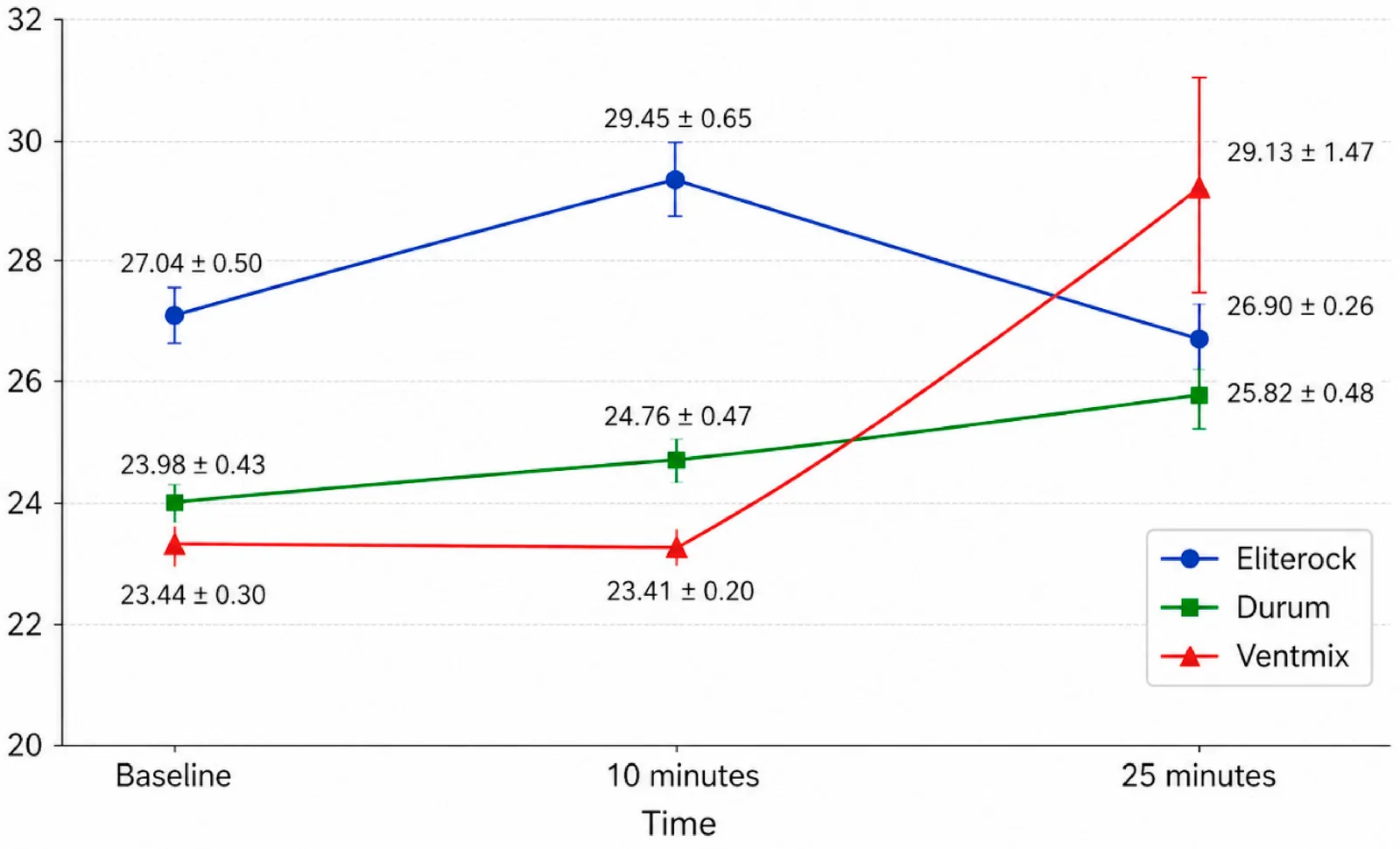
Temperature Values (°C) of Type IV Dental Gypsum according to Time and Brand.

**Figure 2:**
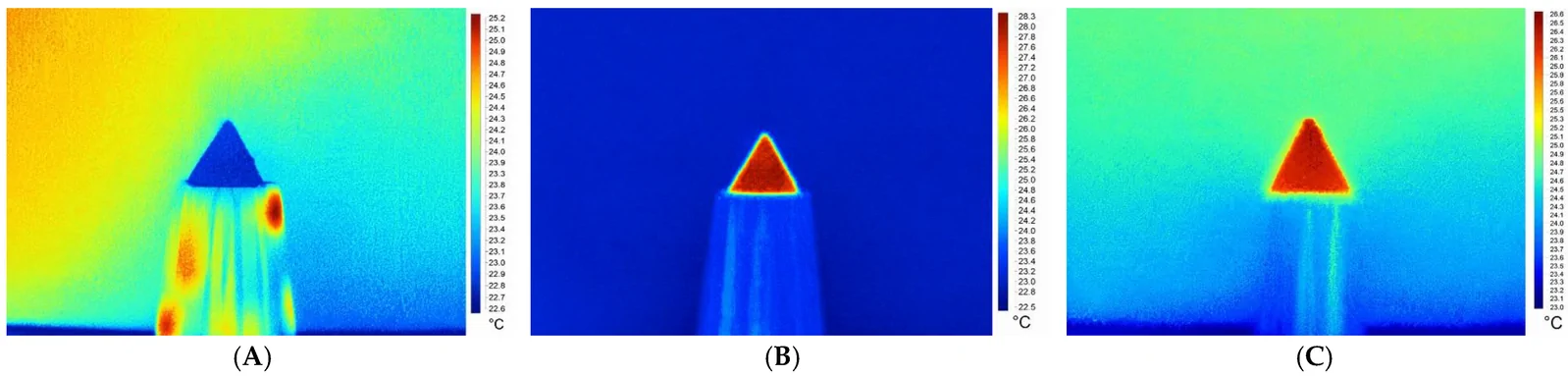
Thermographic evaluation of heat released from type IV dental gypsum. Images corresponding to the final setting measurement (25 min). (**A**): Elite Rock; (**B**): Durum; (**C**): Ventmix.

Values (°C) were significantly different among brands and times (*p* < 0.001). At 10 min after placement, Elite Rock had the largest temperature increase (29.45 ± 0.65 °C), followed by a decrease at 25 min (26.90 ± 0.26 °C). Dürum had a gradual increase from the starting level (23.98 ± 0.43 °C) to 25 min (25.82 ± 0.48 °C). Velmix was relatively stable in temperature from the starting level to 10 min (23.41 ± 0.20 °C), then had a dramatic increase at 25 min (29.13 ± 1.47 °C) (Figure 1). Of the four groups, only the 10 min evaluations were normally distributed; the rest did not pass the Shapiro–Wilk test (*p* < 0.05). The Kruskal–Wallis test produced a statistically significant *p*-value (<0.001) comparing the three types of dental gypsum (Elite Rock, Dürum, and Velmix) and indicated significant differences in the temperature readings across the brands for the three periods (Table 2) (Figure 2).

As the final temperature of Type IV Dental Gypsum increased, it was found that for each minute increment, a corresponding 1.05 °C increase was observed (95% confidence interval [CI]: 0.23–1.87; *p* = 0.012), which reached statistical significance. At 25 min, the temperature increased again to 2.46 °C (95% CI: 1.64–3.28; *p* < 0.001), which is also statistically significant. When compared to Elite Rock (the reference brand), the final temperature of Dürum was significantly lower by −2.94 °C (95% CI: −3.76 to −2.12; *p* < 0.001), and Velmix was reduced by −2.47 °C (95% CI: −3.29 to −1.65; *p* < 0.001). The regression constant for the initial temperature of Elite Rock at time zero was 26.62 °C (95% CI: 25.88–27.37; *p* < 0.001) (Table 3).

**Figure 3:**
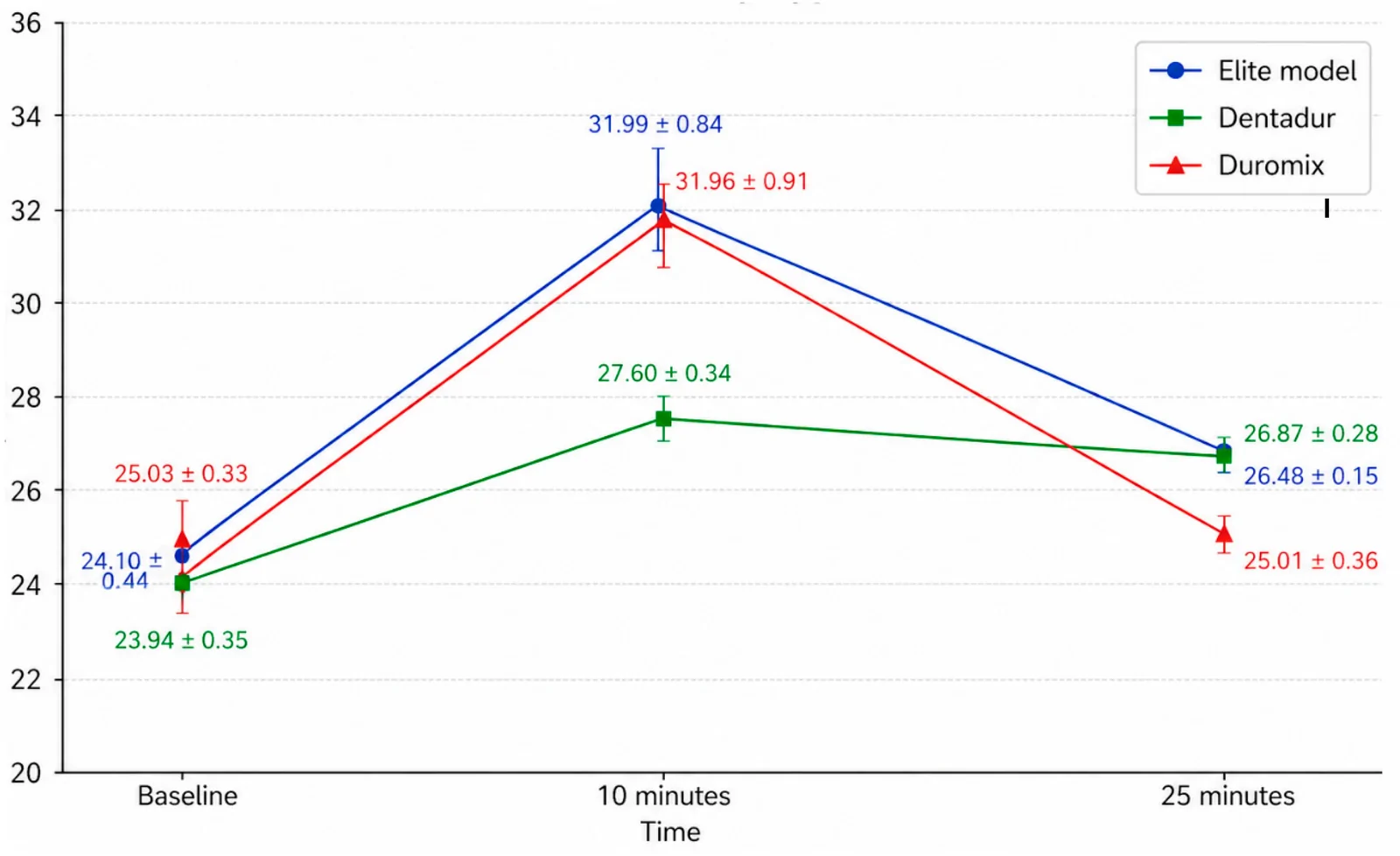
Temperature values (°C) of Type III Dental Gypsum according to time and brand.

**Figure 4:**
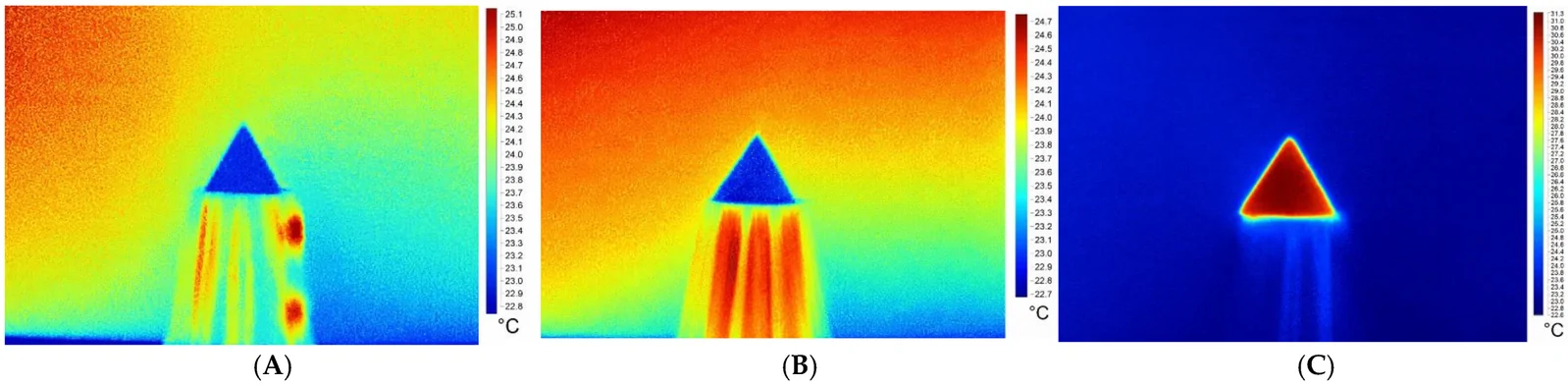
Thermographic evaluation of heat released from type III dental gypsum. Images corresponding to the final setting measurement (25 min). (**A**): Elite model; (**B**): Pentadur; (**C**): Duromix.

At 10 min post-application, the Elite model demonstrated the greatest amount of temperature change, starting with 24.10 ± 0.44 °C at baseline, rising to 31.99 ± 0.84 °C at 10 min, and then decreasing again to 26.48 ± 0.15 °C at 25 min (*p* < 0.001). Pentadur had less drastic changes than Elite, increasing from 23.94 ± 0.35 °C at baseline and reaching 27.60 ± 0.34 °C at 10 min with 26.87 ± 0.28 °C remaining stable for the next 25 min (*p* < 0.001). Both Duromix materials followed the same temperature response as the Elite model, where it quickly increased from 25.03 ± 0.33 °C to 31.96 ± 0.91 °C at 10 min, returning back to its baseline level of 25.01 ± 0.36 °C at 25 min (*p* < 0.001). (Figure 3) There were statistically significant differences between the temperature values between baseline and at 25 min (*p* < 0.001). However, there were no statistical differences between the brands’ values in 10 min (*p* = 0.823). Only the group measured after 10 min had a normal distribution; all other groups were abnormal according to the Shapiro–Wilk test (*p* < 0.05). The Kruskal–Wallis test performed when comparing the three types of dental gypsum (Elite Model, Pentadur, and Duromix) had a *p*-value of 0.823; therefore, no statistically significant temperature differences existed between the brands at the specified time point (Table 4) (Figure 4).

At the 10 min mark, Final Type III Dental Gypsum exhibited a statistically significant rise of 6.16 °C over baseline (95% CI: 5.50–6.82; *p* < 0.001). At the 25 min mark, the increase was more moderate (1.76 °C) but still highly statistically significant (95% CI: 1.10–2.42; *p* < 0.001). The final temperature of Pentadur relative to Elite (the reference material) was significantly lower by 1.39 °C (95% CI: −2.05 to −0.73; *p* < 0.001). In contrast, there was no significant difference between Duromix and Elite with a coefficient of −0.19 °C (95% CI: −0.85 to 0.47; *p* = 0.569). The regression constant (baseline) was 24.88 °C (95% CI: 24.28–25.48; *p* < 0.001) (Table 5).

## 4. Discussion

The study showed that there were differences in surface temperatures recorded by the different brands and types of dental gypsum at the times when they were measured. The regression analyses also indicated that there were differences in surface temperatures as a function of time and the material evaluated. In the case of the Type IV dental gypsum materials, the same general trends were noted for the temperature from all three groups of samples at all three measuring intervals; however, there was a distinct difference in Elite Rock when compared to the other types. Elite Rock had a temperature at 10 min that was higher than the baseline temperature of that material and a temperature at 25 min that was lower than the 10 min temperature. Dürum had a progressively increasing temperature for each of the time periods measured, while Velmix had nearly the same temperature as the baseline and at 10 min, but then had an increased temperature at the 25 min time point. The findings of this study indicate that the temperature changes were different for each of the experimental materials tested during the time intervals measured. As a result of measuring only at baseline, 10 min and 25 min, the present study does not permit conclusions about the maximum temperature for each of the materials tested or the specific time for reaching the maximum temperature for each of the materials tested.

Similar to the findings of the Type III dental gypsum materials, the measured surface temperature of the various evaluated materials also differed over time. While both the Elite Model and Duromix demonstrated higher observed temperatures at 10 min than at baseline, they both had lower observed temperatures at 25 min than at 10 min. Conversely, Pentadur displayed increased surface temperatures at 10 min compared to baseline, but showed a comparatively smaller change in temperature between the 10 and 25 min time points. Therefore, it should be noted that these temperature differences represent point-in-time measurements rather than complete thermal profiles including maximum temperatures and long-term stabilisation of surface temperatures for the evaluated products. Additionally, the updated statistical evaluations that incorporate the time × brand interaction and model-based contrasts should be utilized to assess the differences in changes over time for each of the evaluated products.

Infrared thermography is an objective and non-invasive technique that allows temperature measurements to be obtained without direct contact with the material under evaluation. This approach is consistent with the study by Aboushady et al. [26], which explored the potential application of thermography for assessing periapical inflammatory lesions and demonstrated its utility as a quantitative method for detecting temperature differences. Similarly, Tsuzuki et al. [27] highlighted the potential of thermographic assessment for evaluating temperature changes associated with the chemical reactions of glass ionomer restorative cements. In that study, measurements were obtained at short time intervals, allowing the temporal evolution of the thermal response to be characterized from the early stages of the reaction. Although these studies evaluated different dental applications and materials, they support the potential utility of infrared thermography for investigating temperature changes associated with physicochemical processes in dental materials.

Temperature changes in dental materials are influenced by the progression of physicochemical reactions over time. In the present study, Elite Rock (Type IV) and Elite Model (Type III) showed higher observed temperatures at the 10 min evaluation than at baseline, followed by lower temperatures at 25 min. Although this pattern indicates changes in surface temperature during the evaluated setting period, the discrete measurement schedule does not allow the exact timing or magnitude of the maximum temperature to be determined. Previous thermographic studies have demonstrated that temperature changes associated with dental procedures and material reactions may occur over relatively short periods and can vary according to the material and experimental conditions. Suassuna et al. [28] used thermography to evaluate temperature changes associated with different endodontic filling techniques, whereas Yang et al. [29] demonstrated that the temperature distribution of bulk-fill composite resins changes during polymerization. These findings support the general concept that thermal behavior is time-dependent; however, direct comparisons with the present results should be made cautiously because the materials, chemical reactions, and temporal measurement protocols differ among studies.

This study evaluated surface temperature differences among six commercially available dental gypsum materials at predefined stages of the setting reaction. The observed temperature values differed according to the material and evaluation time, indicating that the thermal response recorded during the setting process was not uniform across the tested products. The revised factorial regression analysis, including the time × brand interaction, was used to determine whether temporal changes differed among materials and to estimate relevant within-material and between-material contrasts. Previous investigations have also demonstrated that the physical and thermal properties of dental materials may vary among commercially available products. Killedar et al. [30] compared different Type IV dental gypsum materials with respect to abrasion resistance under different drying conditions, whereas Bae et al. [31] demonstrated differences in thermal behavior among composite resin materials. Although these studies investigated different outcomes and experimental conditions, they support the broader concept that material composition and product-specific characteristics may influence their physical behavior.

The significance of this study for the scientific community lies in its contribution of evidence on a topic that has yet to be fully explored: temperature variation during the setting process of dental impression gypsum. It also provides a baseline for future research focused on the thermal behavior of these materials and the application of infrared thermography as a noninvasive assessment tool in dentistry. This issue is critically relevant from a clinical standpoint due to the significant role that gypsum models play across many facets of dental treatment. There are different factors that affect how well-suited gypsum models are in a particular case. These factors include hydration, expansion, setting, and drying. Note that all these processes can be directly impacted by the temperature at which the dental gypsum is mixed, applied, and set. Infrared thermography was thus used to objectively assess the differences in temperature associated with the different types of dental gypsum used in this study. The assessment of these thermal characteristics provides valuable insight into the thermal properties of dental gypsum and provides a stronger scientific basis for a dental material discipline that has been relatively limited in scientific information to date [28,29,31].

Differences in the surface temperature values observed among the evaluated dental gypsum materials may be related, at least in part, to variations in the kinetics of the exothermic hydration reaction of calcium sulfate hemihydrate. Factors such as the characteristics of the hemihydrate, particle size and morphology, and differences in the composition or additives of commercial formulations may influence the setting reaction and heat dissipation. However, these physicochemical characteristics were not directly measured in the present study. Therefore, these potential mechanisms should be interpreted as plausible explanations rather than direct causal conclusions derived from the experimental data.

The temperature differences between Elite Rock and Velmix at the surface could also be due to unequal chemical kinetics of their respective setting reactions or from the differences in their commercial products. The temperature of Elite Rock was higher at the 10 min reading compared to that of Velmix, which had its highest value at the 25 min reading, but with the available temperature data points being taken only at the baseline, 10 min, and 25 min, the actual maximum temperature or when the maximum temperature occurred cannot be known. The way the products are made, including the hemihydrate form of the products as well as other proprietary components may affect the heat generated during the chemical reaction and heat release upon hardening of each material, but those factors have not yet been examined as reasons for the differences observed [28,29,30,31,32,33].

There are many limitations of this study that must be accounted for when interpreting the findings. First, the temporal resolution is limited as only three time points were evaluated: baseline, 10 min, and 25 min; therefore, there is no way to continually monitor how the thermal responses changed throughout the contact reaction with thermographic evaluation. Therefore, we could not ascertain both the highest temperature achieved and when that temperature occurred. When interpreting the data, it is important to consider that the values reflect only surface temperatures taken at the points indicated, and not complete thermal profiles. Therefore, it will be important for future studies to include continuous or more frequent temperature readings throughout the setting reaction to allow for determining how the temperature changes develop over time and at what point the maximum temperature occurs. Another limitation of the study is that it evaluated only six commercially available dental gypsum materials, meaning that the results cannot be generalized to all dental gypsum products. Furthermore, even though the experiments were carried out under control in vitro conditions, those conditions do not resemble completely the conditions to which dental gypsum products are exposed in clinical or laboratory settings. Last, the infrared thermography only provided surface temperature readings on the specimen; there was no additional information concerning temperature changes throughout the mass of the material. Subsequent research should include more diverse products and more varied environmental conditions, as well as conduct studies that use infrared thermography along with other techniques for monitoring the internal temperature changes.

A key strength of this study is the application of infrared thermography as an objective non-invasive technique to assess temperature without changing the chemical and/or physical properties (characteristics) of the materials being evaluated. Additionally, this study used commercially available brands of dental gypsum most used in routine dental practice, thus making the results more clinically relevant. The use of linear regression allowed accurate quantification of temperature variation based on evaluation time, brand analysis, and provides an opportunity for more accurate interpretation of thermal results observed during the dental gypsum setting protocol. In addition, through examination of an area that has been largely unexplored in the scientific literature to date, this research provides valuable contributions to the body of knowledge within a scientific area with minimal academic output. Further, it is hoped that the results presented here could lead to further research into the thermophysiological properties of dental gypsum.

## 5. Conclusions

The findings of this in vitro study demonstrate that the surface temperature values of the evaluated dental gypsum materials varied significantly from material to material and over time. The temperature variation demonstrated in this study, when measured at three discrete time points (baseline, 10 and 25 min) were not comparable as to the total increase and decrease in temperature during the setting process. Infrared thermography was utilized in this study as an effective non-invasive method to quantify the surface temperature variations during the setting process. As such, it is important that additional studies utilizing a continuous or high-frequency monitoring system be conducted in order to characterize the complete thermal response of dental gypsum materials to provide a better understanding of how thermal responses correlate with other physicochemical properties of the materials, including dimensional stability and performance.

## Figures and Tables

**Table 1:** Types of dental gypsum.

Brand	Type	Country	Features	Ingredients	Batch	Preparation
Zhermack–Elite Rock Fast	Type IV	Zhermack S.p.A., Badia Polesine (RO), Italy	High hardness, low expansion, thixotropic, ideal for fixed prostheses	Modified calcium sulfate hemihydrate (α)	466504	20 mL water/100 g; mix for 30–60 s; work for 5 min; set for 9 min.
Ventmix	Type IV	Lima, Peru	Good strength, low expansion, for dies	Calcium sulfate hemihydrate (α)	0405-29578	22 mL water/100 g; mix for 1 min; work for 8 min; and set for 11 min.
Durum (MDC Dental)	Type IV	MDC Dental, Mexico	High abrasion resistance, high hardness, low expansion, thixotropic	Modified calcium sulfate (α) + additives	P250909277	20 mL water/100 g; mechanical mixing 20–30 s/manual mixing 30–60 s; working time 5–7 min; setting time 7–10 min
Zhermack–Elite Model Fast	Type III	Zhermack S.p.A., Badia Polesine (RO), Italy	Good strength, thixotropic, general-purpose	Improved calcium sulfate hemihydrate (β)	481047	30 mL water/100 g; mixing 30–60 s; working time 4 min; setting time 8 min
Duromix	Type III	Lima, Peru	Smooth surface, good precision, medium hardness	Calcium sulfate hemihydrate (β)	25-01600	28–30 mL water/100 g; mix 60 s; work 5 min; set for 12 min
Pentadur	Type III	Lima, Peru	General-purpose, moderate strength, and economical	Calcium sulfate hemihydrate (β)	Without registration	32–35 mL water/100 g; mix manually 60 s/mechanically 40–50 s; work 5 min; initial setting 4–5 min

**Table 2:** Temperature values (°C) in Type IV gypsum according to brand and time.

Type IV Dental Gypsum	Baseline	10 min	25 min	*p* *
Elite Rock	27.04 ± 0.50	29.45 ± 0.65	26.90 ± 0.26	
Dürum	23.98 ± 0.43	24.76 ± 0.47	25.82 ± 0.48	<0.001
Velmix	23.44 ± 0.30	23.41 ± 0.20	29.13 ± 1.47	
*p* *		<0.001		

* Kruskal–Wallis test. None of the groups presented a normal distribution, as the Shapiro–Wilk test yielded *p*-values < 0.05 in all cases.

**Table 3:** Linear regression model for the final temperature of type IV dental gypsum according to time and brand.

Variable	Coefficient (β)	*p*	CI 95%
Time (ref: Baseline)			
10 min	1.05	0.012	0.23 a 1.87
25 min	2.46	<0.001	1.64 a 3.28
Gypsum (ref: Elite Rock)			
Dürum	−2.94	<0.001	−3.76 a −2.12
Velmix	−2.47	<0.001	−3.29 a −1.65
Constant	26.62	<0.001	25.88 a 27.37

**Table 4:** Temperature values (°C) in Type III gypsum according to brand and time.

Type III Dental Gypsum	Baseline	10 min	25 min	*p* †
Elite Model	24.10 ± 0.44	31.99 ± 0.84	26.48 ± 0.15	
Pentadur	23.94 ± 0.35	27.60 ± 0.34	26.87 ± 0.28	<0.001
Duromix	25.03 ± 0.33	31.96 ± 0.91	25.01 ± 0.36	
*p* †		0.823		

† Kruskal–Wallis Test. Only the group evaluated at 10 min presented a normal distribution, whereas all other groups did not, as the Shapiro–Wilk test yielded *p*-values < 0.05.

**Table 5:** Linear regression model for the final temperature of type III dental gypsum according to time and brand.

Variable	Coefficient (β)	*p*	IC 95%
Time (ref: Baseline)			
10 min	6.16	<0.001	5.50 a 6.82
25 min	1.76	<0.001	1.10 a 2.42
Dental Gypsum (ref: Elite model)			
Pentadur	−1.39	<0.001	−2.05 a −0.73
Duromix	−0.19	0.569	−0.85 a 0.47
Constant	24.88	<0.001	24.28 a 25.48

## Data Availability

The datasets generated and analyzed during the current research are available from the corresponding author upon reasonable request.
